# Bibliographical Notices

**Published:** 1853-12

**Authors:** 


### BIBLIOGRAPHICAL NOTICES.

*La Syphilisation étudiée comme méthode curative et comme moyen prophylactique des maladies vénériénnes, par* C. SPERINO, Agregé a la Faculté Médico-Chirurgicale de Turin etc. Paris, 1853.

*Syphilization studied as a Curative and Prophylactic measure in Venereal Diseases, etc.*

No experiments have excited more interest amongst professional men, than those recently performed in Europe, to test the value of syphilization.

Without discussing the question whether syphilis was known to the ancients or not, we may state that an accurate knowledge of venereal diseases cannot be traced back further than John Hunter. His experiments, indeed, repeated and modified by Ricord, have led to the adoption of a system which has served as a basis for the diagnosis and treatment of all venereal diseases. The most important points Ricord established were:

1. That chancre and blennorhagia are two distinct affections.
2. That only indurated chancres are followed by constitutional syphilis.
3. That only indurated chancres require a constitutional treatment.
4. That secondary affections are not inoculable.
5. That syphilis cannot be transmitted to animals.

Experiments performed by M. Auzias-Turenne, to test the truth of this last proposition, led him to the observation of the curious fact, that each successive chancre inoculated is smaller than its predecessor. Several inoculations performed on a medical student seemed to be followed by the same result; and these gave rise to the doctrine of "syphilization," or the inoculation of syphilitic virus as a prophylactic measure against syphilis, and as a therapeutical agent for its cure. "If," says M. Auzias-Turenne, "each successive chancre is smaller than its predecessor, must we not ultimately arrive at a point in which the constitution is so impregnated with syphilitic virus as to be incapable of receiving new chancres ?" An individual who has arrived at this point of syphilitic "saturation" he called *syphilized ;* the process itself, syphilitic vaccination or *syphilization.*

Had the means of cure hitherto possessed, been found adequate to the treatment of all syphilitic diseases, syphilization would have attracted, at best, but a passing attention. But every one knows how many old syphilitic affections prove refractory under the usual method of treatment, and, therefore, every new agent proposed is always eagerly tried. The hope, too, of acquiring celebrity by discovering a new remedy for syphilis, excited many to enter upon the field of experimentation, whilst others were induced by the controversy to repeat Ricord's experiments on inoculation, with the view of testing their value. Thus originated the experiments on inoculation and the discussions on syphilization, which have so recently been agitating the profession abroad.

Amongst those who, without entering into the subject of syphilization, confined their experiments to inoculation, we may mention Prof. Waller, of Prague,* M. Vidal de Cassis,† M. Maisoneuve ;‡ amongst those who made use of syphilization on patients, M. Auzias Turenne, M. Diday, of Lyons, and M. Sperino, of Turin. This latter observer, indeed, has recently published a complete work on the subject, containing not only all that up to the time had been written on syphilization, but also a report of ninety-six cases, in which this method was employed with great success. M. Sperino's work occupies over 800 pages, chiefly made up of the narration of cases. Of its contents, therefore, we can give but the merest sketch.

The author states in his preface, that his experiments were performed with the greatest care and precision, and the results

* Ueber den contagiöesen character der secundaeren syphilis; Prague, 1851.

† Traité des Maladies Vênériennes ; Paris, 1853.

‡ Traité pratique des Maladies Vénériennes. Par G. Maisoneuve, et Montonier ; Paris, 1853.

Matter bearing on this subject will further be found in the Report of the Paris Commissions to the Prefect of Police. (Report á M. le Préfet de Police sur la question de savoir si M. le Dr. Auzias-Turenne peut être autorisé à appliquer la Syphilization á l'infirmerie de la Prison St. Lazars ? par M. M. Melier, Ricord, Denis, Conneau et Marchal de Calais), and in Ricord's letter on Syphilis (Lettres sur la Syphilis; Paris, 1852.)

given with the loyalty of a man of honor. We are bound to believe him, although some of his facts seem almost incredible. He commences his work with a consideration of the value of inoculation, as applied to venereal diseases. Inoculation, he says, has been recently practised for seven different purposes:

1. To prove the existence of a syphilitic virus.
2. To distinguish true from false syphilitic diseases.
3. To establish the differences which exist between primary and secondary syphilitic affections.
4. To prove the efficacy of certain therapeutical agents called preservatives.
5. To ascertain if syphilitic diseases could be communicated to animals.
6. As a therapeutical measure against syphilis.
7. As a prophylactic agent against this disease.

We pass over the first five propositions (mentioning only that M. Sperino denies the difference between syphilis and blennorhagia) and revert to propositions six and seven, or the value he attaches to inoculation as a prophylactic and therapeutical agent. Speaking of its efficacy he says in his Report to the Academy at Turin:

"All recent chancres existing in women who were subjected to this treatment disappeared spontaneously a few days after the appearance of the artificial chancres. Indurated syphilitic ulcerations which had existed for two or four years, and had resisted a treatment by mercury, by iodide of potassium, or even by the acid nitrate of mercury, or the Vienna paste, cicatrized rapidly after a few inoculations with pus taken from the recent chancres of other women. Large and deeply rooted virulent ulcerations cicatrised a short time after inoculation, and, what is more astonishing, I saw in a woman who had a chancre and two inguinal buboes, in both of which, distinct fluctuation could be felt, the pus absorbed and disappear in a few days after the inoculation on the abdomen of several artificial chancres. Mucous tubercles existing at the same time with the chancres, disappeared under the powerful action of inoculated virus. A large ulcer situated on the posterior portion of the pharynx, healed up after the 5th inoculation. In one woman herpiginous ulcers having their seat on the right leg cicatrized rapidly, whilst, at the same time, the pains in the frontal bone disappeared after the development of the artificial chancres.

The harmlessness and the advantage of syphilitic inoculation, became at last so well known to the patients, that they themselves, forgetting their reluctance to the first trials, begged me to syphilise them, as their companions had been thus cured."

The method of producing the artificial chancres consists in puncturing the skin with a lancet, on which venereal matter has been collected. The place best suited is the thigh or abdomen. The chancres thus produced do not differ in appearance from ordinary chancres, except that, after repeated inoculations, they become smaller. The number of inoculations necessary to produce syphilization we do not find mentioned; in some cases five, in others sixty or more seem to have been necessary. The cicatrices of these artificial chancres are very superficial, except when they become gangrenous. During the treatment by syphilization, baths and purgatives were freely employed; the cicatrices were dressed with simple cerate.

Now follows the report of ninety-six cases cured by syphilization. To attempt a full analysis of these cases would carry us far beyond the limits assigned to a review; we therefore select but one of the most striking and interesting. (Observation I, p. 116.)

"Julia P., age 22, of a sanguine-lymphatic temperament and excellent constitution, entered the hospital on the 29th of January, 1851.—She has on her body several chancres, viz., an urethral chancre, evidently indurated, a chancre in the fossa navicularis in size about 12 millimetres and also indurated, and two large and irregular chancres situated on the sides of the urethra. It is the second syphilitic infection under which she labors. In 1845 she had an indurated chancre on the vulva, accompanied with a bubo in the groin, and mucous tubercles around the arms and vulva. She was then treated with 40 mercurial inunctions. Constantly returning vegetations obliged her to remain in the hospital during the whole of the year 1846. As excision and cauterization did not prevent their reproduction, I subjected her to a new treatment by inunction. But even this did not triumph over the "vegetative" power of the vulvo-vaginal mucous membrane. The vegetations finally yielded to deep incisions and repeated cauterizations. Since 1845 up to this day, she has been sent nine times to the hospital with chancres, twice complicated with urethral blennorhagia, and which have always been cured by a simple local treatment.

*20th of January*, 1851, (the day she entered the hospital,) an inoculation was made on her left thigh, with the pus taken from her other chancres; a large pustule was the result, which soon changed into a syphilitic ulcer; the inoculation was repeated on the 31st, and another pustule was the result.

*27th of February.*—These two artificial chancres are cicatrized. Their size is not over eight millimetres. Three of the chancres on the vulva are in progress of cicatrization, and have diminished already considerably—namely, the one on the fossa navicularis and the two occupying the side of the urethra. The one situated on the canal and the orifice of the urethra is still virulent, as three inoculations made with the pus it secretes produced three pustules."

*3d of March.* Two more inoculations which were repeated on the 10th and 13th, with the pus taken from artificial chancres of another woman. We have now altogether 6 chancres produced by inoculation.

*20th of March.* The 6 chancres produced from the three last inoculations are still open; their size varies between three and six millimetres. They are very superficial.

The chancres on the sides of the urethra, as well as the one in the fossa navicularis, have headed. The urethral chancre is in process of granulation, but its induration still persists. Four more inoculations made with the pus taken from the chancre of another woman, who had just entered the hospital, produced no effects.

*3d of April.* Four more inoculations; no result.

*10th of April.* All the artificial chancres are cicatrized.

*21st of April.* Three more inoculations; three more chancres.

*28th of April.* Two more inoculations; no result.

*29th of May.* Three more inoculations on abdomen, and two on the internal surface of the nymphæ; no result.

*29th of June.* Julia P. has been received into the hospital as a nurse. The induration of the urethra has not completely disappeared. Some fungosities still remain in the urethral canal; they are touched from time to time with the nitrate of silver. It is thought advisable to continue from time to time the inoculations with the view of producing a complete syphilization. On the seventh of July two new inoculations without result. On the twentieth of July, six new inoculations without without result.

*31st of December*, 1852. Julia P. is still in the hospital as a nurse. Her health is excellent, and she has never had the slightest symptoms of constitutional infection. In conclusion I may remark, that in this case I observed two indurated chancres separated from each other by long intervals of time. The first appeared under the use of mercurials; the second by syphilization. I know that this case will find many incredulous persons amongst the followers of Ricord, but truth should triumph over all theories."

This is certainly a case which, if correctly stated, would tend to prove the efficacy of syphilization. If the second chancre was really indurated, it ought, according to the doctrines of Ricord, to have been followed by a constitutional syphilis at furthest within six months after the primary affection; whilst M. Sperino had an opportunity of examining the patient for nearly two years, and could observe no secondary symptons.

We might add several more of these cases, and especially those of inveterate constitutional syphilis, in which this treatment seems to have been employed with great success, (see pp. 248, 337, 475,) but we can here but notice the principal facts they contain, which are mostly in direct opposition to the doctrines of Ricord, and may be grouped together as follows:

1. Indurated chancres are often followed by suppurating buboes.
2. Non-indurated chancres may be followed by constitutional syphilis.
3. Blennorhagia may give rise to constitutional syphilis.
4. Syphilization will cure when mercurials and Iodide of Potassium fails.

Out of the ninety-six cases Mr. Sperino reports, we have fifty-three affected with primary, and forty-three with constitutional syphilis. Of these fifty-three cases of primary syphilis, fifty were treated by syphilization, and only in two the treatment was unsuccessful. In one case mercurial treatment was employed at the same time. Out of the forty-three cases of constitutional syphilis, six were treated by syphilization and the iodide of potassium; eight by means of syphilization, iodide of potassium, and mercury; two died, leaving twenty-three cases cured by syphilization only. A careful examination of the history of the patients affected with primary syphilis will show that a great many had non-indurated chancres, which probably would not have given rise to any constitutional syphilis, and which would have recovered under a simple local treatment. In the cases of secondary syphilis, syphilization seems to have proved singularly efficacious, and yet the occurrence of phagedenic ulcers after inoculations, (a circumstance which M. Sperino himself admits,) makes even here the remedy but little less dangerous than the disease. The time employed for this treatment varies: one case was cured in less than a month; four in one to three months; seven, three to six months; eight, six to nine months; eight, nine to twelve months; twenty-one, twelve to fifteen months; whilst twenty-seven required from fifteen to seventeen months. A much longer period than would tend to prove the efficacy of syphilization by any of the ordinary treatments.

With these tables of the time employed, M. Sperino terminates the most important part of his work. The succeeding chapters are lengthy and tedious. They consist mainly in a continued repetition of what has already been said, and in an abuse of Ricord and his followers. M. Sperino toils, too, to prove that in all cases in which syphilization did not succeed after repeated inoculations, it could be attributed to some fault in inoculating. He endeavors, indeed, to show that the celebrated case of Dr. L——* who after more than two hundred inoculations, was not yet syphilized, could be attributed to this. We will not follow M. Sperino in the terminal chapter of his book, which are devoted to an analysis of all the writings on syphilization, and to a correspondence between himself and several syphilographs. The most important conclusions M. Sperino arrived at, he himself states to be:

1. Syphilization will cause the symptoms of primary syphilis to disappear.

2. Syphilization, instead of being rejected as a prophylactic measure of primary syphilis, ought, on the contrary, to be carefully studied in this point of view, as we have but a small number of individuals completely syphilized, who have contracted a new infection.

3. Syphilization ought, as much as possible, to be limited to the treatment of constitutional syphilis until we are certain that syphilization prevents the syphilized from contracting a new infection.

With these general deductions the work of M. Sperino terminates. As to the truth of the principles on which the doctrines it professes are based, we refrain from giving an opinion. A work containing a report of cases and with the motto *non verbis, sed factis*, can only be refuted with a sufficient number of facts demonstrating the contrary. Whether repeated inoculations make it impossible for new chancres to be contracted, future researches will show. We have now already some observations (as the one of Dr. L——,) which will tend to show the fallacy of even this view. As a therapeutical measure we do not see, even if the statement of the cases be correct, any advantage syphilization possesses in efficacy or in shortness of duration over the remedies we at present employ. As a prophylactic measure it

*Dr. L—— was presented to the Academy in Paris, in June, 1853, by M. Ricord, and a report read on his case.

is highly dangerous and demoralizing, and we agree with Ricord in his exclamation at the Academy:

"If, after all, syphilization, as it is *at present* presented to us, should be true, it ought, nevertheless, to be legally prohibited as a prophylactic measure, and rejected as a treatment."

---

*Lectures on Surgical Pathology, delivered at the Royal College of Surgeons of England.* By JAMES PAGET, F. R. S., late Professor of Anatomy and Surgery to the College, Assistant Surgeon and Lecturer on Physiology at St. Bartholomew's Hospital. Philadelphia. Lindsay & Blakiston, 1854. 8vo. pp. (with index) 699.

This book consists of a series of lectures delivered by Mr. Paget, a few years since, in the chair of Anatomy and Surgery in the Royal College of Surgeons of England. Some of them have already appeared in other forms. But their popularity has happily induced the author to amplify and revise them, and present them in this shape to the profession.

The title of the work is hardly expressive of its scope and value. It is, indeed, nominally restricted to the pathology of diseases which belong, in treatment, to the domain of surgery rather than of medicine. But it is really illustrative of the general subject of pathology, and will be read with equal interest and instruction by both the physician and the surgeon.

Mr. Paget has long been ranked among the foremost minds of the profession in Great Britain. And his pursuits have perhaps especially fitted him for such a subject as that of this book. "Engaged in the simultaneous practice of surgery and teaching in physiology,"—formerly Professor of Anatomy and Surgery, and now Lecturer on Physiology—he has certainly enjoyed particular advantages for "illustrating the general pathology of the principal surgical diseases, in conformity with the larger and more exact doctrines of physiology." These advantages have been admirably turned to account in the masterly work which now commends itself to the notice of the American profession.

The subjects treated in these lectures are the following:—1. Nutrition, its nature, purpose, and conditions. 2. The conditions necessary to healthy nutrition. 3. The formative process; Growth. 4. Hypertrophy. 5. Atrophy; Degeneration.

6. Atrophy. 7. General considerations on Repair and Reproduction. 8. The Materials for the Repair of Injuries. 9 and 10. The process of the Repair of Wounds. 11. The Repair of Fractures. 12. The Repair of Injuries in various tissues. 13. Phenomena of Inflammation. 14. Products of Inflammation. 15. Developments of Lymph. 16. Degeneration of Lymph. 17. Changes produced by Inflammation in the affected part. 18. Nature and Causes of Inflammation. 19. Mortification. 20. Specific Diseases. 21. Classification of Tumours. 22. Simple or Barren Cysts. 23. Compound or Proliferous Cysts. 24. Fatty and Fibro-Cellular Tumours, Painful Subcutaneous Tumours. 25. Fibrous Tumours. 26. Cartilage Recurring Fibroid and Fibro-nucleated Tumours. 27. Cartilaginous Tumours. 28. Myeloid and Osseous Tumours. 29. Glandular, and Vascular or Erectile Tumours. 30. Scirrhous or Hard Cancer. 31. Medullary Cancer. 32. Epithelial Cancer. 33. Melanoid, Hæmatoid, Osteoid, Villous, and Colloid Cancers. 34. General Pathology of Cancer. 35. Tubercle.

We cannot attempt to follow Mr. Paget through these subjects in detail. His views, however, on the highly interesting subjects of the origin, progress, and products of inflammation, deserve to be presented somewhat in extenso.

Mr. Paget thus sums up his conclusions as to the circumstances which may determine the characters of an inflammatory exudation to be adhesive or suppurative.

"To sum up, then, what may be concluded respecting the conditions that, in the first instance, may determine the adhesive or suppurative characters of an inflammatory exudation; they are, 1*st*, The state of the blood,—its diathesis or crasis,—the power of which is evident in that the same material may be exuded in many inflamed parts in the same person; in that this material may exhibit peculiar characters correspondent with those of the blood itself; and in that, in different persons, an inflammation excited in the same tissue, and by the same stimulus, will produce different forms of lymph, corresponding with differences of the blood. 2*d*, The seat of the inflammation, and the tissue or organ affected; of which the influence is shewn by cases in which, with the condition of blood, different forms of lymph are produced in different parts or organs. 3*d*, The severity, and acute or chronic character, of the inflammatory process, according to which the product deviates more or less from the character of the natural secretion or blastematous effusion in the part.

"The primitive character or tendency of any case of inflammation might be represented as the resultant of three forces issuing from these conditions."

As regards the products of inflammatory exudation, we find the following remarks :

"*Fibrous Tissue*, as the result of the development of lymph, is found when the exudation is interstitial in any fibrous tissue ; as in ligaments, capsules of joints, and the like. The best examples of it are in the laminated and nodular thickenings of the capsule of the spleen, or the thickening and induration of the periosteum, or the capsule of the hip-joint in chronic rheumatic arthritis. In all these cases the new material is derived from repeated, but not acute, inflammations; therefore, probably, though excessive, it is not widely different from the normal material for nutrition ; and, the conditions for nutrition being little disturbed, it is developed into the exact likeness of the original texture with which it is intermingled and confused.

"As the fibro-cellular and fibrous tissues, formed from inflammatory lymph, become more perfectly organized, they are prone to contract ; imitating the contraction already described in granulations and scars (p. 236). Hence, in part, the contraction of the wall of the chest after pleurisy, and the various displacements and deformities of organs that have become adherent to adjacent parts ; hence, in part also, the contractions of inflamed organs, as of the liver in cirrhosis ; hence, too, an addition to the rigidity of joints when the parts around them have been inflamed ; and hence, with yet greater mischief, the contractions of the thickened valves and tendinous cords of the heart.

"*Adipose Tissue* may be formed, if not directly from inflammatory lymph, yet in the fibro-cellular tissue of completely organized adhesions. I think it is not often so formed ; but, lately, Dr. Kirkes found a lung of which all the anterior part was covered with well-organized false membrane ; and in part of this was a quantity of perfect adipose tissue, more than four ounces in weight.

"*Elastic Tissue* is sometimes abundantly formed in the adhesions developed from inflammatory lymph. I have not seen it except in such as are completely organized ; and I think it is, in this case, as in the formation of scars, a late production. I believe, also, with Virchow, that its formation depends, in some measure, on the membrane that is inflamed ; pleural adhesions being most favorable to it. In these it is often abundant ; its principal, but always slender, filaments lying in the same general direction as those of the fibro-cellular tissue.

"*Epithelium* I have already mentioned as covering the surfaces of well-formed adhesions. I know of no observations proving whether the epithelial cells are developed directly from the lymph, or are a later construction from materials derived from the blood of the adhesion's vessels ; but it is not rare to find, in inflammation of serous membranes, recent lymph-cells presenting many characters indicative of development towards epithelium ; flattened and enlarged, and having circular or oval clear nuclei.

"*Bone* is often formed from inflammatory lymph. It may appear as a late transformation of lymph that has been organized into perfect fibrous tissue; as in the osseous plates that are sometimes found in the false membranes of the pleura, or in the pericardium. In most of these, however, there is not true bone, but an amorphous deposit of earthy matter, which is imbedded in the fibrous tissue, or which (as Rokitansky holds) is the residue of the degenerated and partially absorbed tissue."

Again, on the formation of blood-vessels in effused lymph:

"1. The direct observations supposed to prove that blood is formed in lymph are very liable to fallacy, through the facility with which blood may be accidentally mixed with the lymph, in consequence of hæmorrhage during life or after death, or in the preparation of the specimens. Where these sources of fallacy have been avoided, I have never seen anything suggestive of a transformation of lymph into blood.

"2. The development of blood from tissue-cells is limited, naturally, to the earliest period of embryo-life, as if it needed the greatest amount of force for development; afterwards, blood is not formed except through a long process of elaboration, and with the aid of many organs. Its formation, therefore, in the mal-conditions of inflammation is very improbable.

"3. In no specimen of inflammatory lymph have I seen appearances of transitions from lymph-cells to blood-cells, such as we may see in the lymph of the lymphatics, both before and after it is poured into the blood-vessels.

"4. Neither in any lymph have I seen appearances of such stellate cells as the interstitial bloodvessels of the early embryo are formed from; nothing comparable with them has ever come into view.

"5. In the formation of vessels for granulations and the walls of chronic abscesses, all is favorable to the belief that they grow up from the bloodvessels of the adjacent parts; and there are no structures to which the lymph bears so close analogy as it does to these, or to which it is so likely to be conformed in the production of its vessels.

"On the whole, therefore, although direct observations are wanting, I think we may conclude that all the vessels of inflammatory lymph are formed by outgrowth from adjacent vessels, as in the process of repair, and that through these vessels, not by its own development, it derives its supply of blood."

We conclude these extracts with Mr. Paget's views on the intimate nature of inflammation.

"If we consider the quantity of organic formation effected during the inflammatory process, in the proper substance of the inflamed part, it is evidently less than in health. All the changes described in the last lecture are examples of diminished or suspended nutrition in the tissues of the inflamed part; they are all characteristic of atrophy, degeneration, or death. The tissues become soft, or quite disorganized; they are relaxed and weakened; they degenerate, and remain lowered at once in structure, chemical composition, and functional power; or else, after degeneration, they are absorbed, or are disintegrated, or dissolved, and cast out; they die in particles or in the mass. During all the process of inflammation there is no such thing as an increased formation of the natural structures of the inflamed part; they are not even maintained; their nutrition is always impaired, or quite suspended. It is only after the inflammation has ceased that there is an increased formation in some of the lowly organised tissues, as the bones and cellular tissue.

"So far, then, as the proper substance of the inflamed part is concerned, there appears to be decreased action; that is, decreased formation. There may be, indeed, an increased absorption; but this is also, in one sense, characteristic of decreased exercise of vital force; since all absorption implies a previous degeneration of the part absorbed. Nor can we justly call this, in any sense, 'increased action,' till we can shew how absorption is an action of vessels.

"Thus far, one of the constituents of the inflammatory process, one of the characters in which it differs, in respect of quantity, from normal nutrition, is a defect in the nutrition of the proper substance of the inflamed part.

"But it is characteristic of the complete process of inflammation, that, while the inflamed structure itself suffers deterioration, there is a production of material which may be peculiarly organized. Here, therefore, may be an evidence of increased formation, of increased action.

"Doubtless, in relation to the productive part of the inflammatory process, the expression "increased action" may be in some sense justly used; for the weight of an inflamed part, or of the material separated from it, may be much increased by the formation of organized matter. But the quantity of organized matter formed in an inflammation must not be unconditionally taken as a measure of increase in the exercise of the vital forces; for it is to be observed, that the material formed presents only the lowest grades of organization, and that it is not capable of development, but rather tends to degeneration, so long as the inflammation lasts.

"It may be but a vague estimate that we can make of the amount of force exercised in any act of formation; yet we may be sure that a comparatively small amount is sufficient for the production of low organisms, such as are the fibrinous and corpuscular lymphs of inflammation. The abundant production of lowly organized structures is one of the features of the life of the lowest creatures, in both the vegetable and animal kingdoms. And, in our own cases, a corresponding abundant production is often noticed in the lowest states of vital force; witness the final inflammations, so frequent in the last stages of granular degeneration of the kidneys, of phthisis, of cancer, and other exhausting diseases. In all these, even large quantities of the lowly organized cells of inflammatory lymph may be formed, when life is at its last ebb. And with these cases those correspond which shew the most rapid increase of tubercle and cancer, and of lowly organized tumors, when the health is most enfeebled, and when the blood and all the natural structures are wasting.

"From these considerations we conclude that the productive part of the inflammatory process is not declaratory of the exercise of a large amount of formative or organizing force; and this conclusion is confirmed by observing that development, which always requires the highest and most favored exercise of the powers of organic life, does not occur while inflammation lasts. The general conclusions, therefore, may be, as well from the productive, as from the destructive, effects of the inflammatory process, that it is accomplished with small expenditure of vital force; and that even when large quantities of lymph are slowly organized, such an expression as 'increased action' cannot be rightly used, unless we can be sure that the defect of the formative power, exercised in the proper tissue of the inflamed part, is more than counterbalanced by the excess employed in the production and low organization of lymph.

"It may be said that the signs of inflammation are signs of increased action. But these are fallacious, if again, by increased action be meant any increased exercise of vital force. The redness and the swelling of the inflamed part declare the presence of more blood; but this blood moves slowly; and it is a quick renewal of blood, rather than a large quantity at any time in a part, that is significant of active life. An abundance of blood, with slow movement of it, is not characteristic of activity in a part; it often implies the contrary, as in the erectile tissues, and the cancellous tissue of bones.

"The local increase of heat is too inconstant to afford ground for judging of the nature of inflammation. When manifest, it is not, I think, to be exactly compared with that of an actively growing part, or of one which is the seat of 'determination' of blood, or of 'active conjestion." In these cases the heat is high, chiefly because the blood, brought quickly from the heart, is quickly renewed; but in an inflamed part the blood is not so renewed; it moves more slowly. The heat may, indeed, be in some measure ascribed to this condition; for the quickly moving blood around the inflamed part may communicate its heat to that which is moving more slowly. But the proper heat of inflammation (I mean that which is measureable by the thermometer) cannot, I think, be wholly thus explained. Some of it is probably due to the oxidation of the degenerating tissues; a process which we might safely assume to be rapidly going on in the more destructive inflammations, and which is, indeed, nearly proved by some of the evidences of the increased excretion of oxidized substances in inflammations, especially by the increase of phosphates in the urine during inflammation of the brain. It is far from proved, indeed, that this source of heat is sufficient for the explanation of the increase in an inflamed part; and it may be at once objected that we have no evidence that the hottest inflamed parts are those in which the most destructive processes are going on. Still, in relation to the question, how far the increased heat is a sign of the quantity of formative force that is being exercised, we may argue that, as the general supply of heat in our bodies is derived from oxidation or combustion of wasted tissues or of surplus food, so, in these local augmentations of heat, the source is rather from some similar destruction of organized substances, than from increased formation of them. If it be so, increased heat will give no ground for regarding the inflammatory process as the result of a greater exercise of formative force than is employed in ordinary nutrition ; none for speaking of it as increased nutrition, or increased action. Rather, this sign may be added to the evidences that the inflammatory process presents, of diminished formative force, and of a premature and rapid degeneration, in the affected part."

---

*The Medical Formulary : being a Collection of Prescriptions*, etc., etc. By BENJAMIN ELLIS, M. D., late Professor of Materia Medica in the Philadelphia College of Pharmacy. *Tenth Edition, Revised and much Extended.* By ROBERT P. THOMAS, M. D., Professor of Materia Medica in the Philadelphia College of Pharmacy. Philadelphia, Blanchard & Lea. pp. (with index) 296.

This standard old favorite is here introduced for the *tenth* time to the American profession, under the editorial supervision of Dr. R. P. Thomas. Numerous improvements and additions have been introduced, and "all the formulæ embracing officinal articles have been brought to the standard of the National Pharmacopœia."

---

*Yellow or Malignant Bilious Fever in the Vicinity of South Street Wharf, Philadelphia*, 1853. By WILSON JEWELL, M. D. pp. 40.

Dr. Jewell's valuable and interesting report on the Yellow Fever which prevailed in our city last summer, (as read before the College of Physicians, Aug. 3 and Sept. 7, 1853,) is here presented in pamphlet form. Our readers have already had the benefit of a synopsis of this report in the editorial columns of our journal for September and November. The more detailed history of the Fever, however, as contained in the report, should be generally read.

---

*A Treatise on the Venereal Disease.* By JOHN HUNTER, F. R. S. *With Copious Additions.* By Dr. PHILIP RICORD. *Edited with Notes.* By FREEMAN J. BUMSTEAD, M. D., Physician to the North-Western Dispensary, New York. Philadelphia : Blanchard & Lea, 1853. 8vo. pp. (with index) 520.

This many-headed book presents us the great Treatise of John

Hunter on the Venereal Disease, surrounded with a rather complicated net-work of "notes and additions." The original treatise of Hunter (of the edition of Mr. G. G. Babington, and including the contributions of Sir Everard Home,) was published in Paris in 1840, with annotations by M. Ricord. The volume before us consists of a translation by Dr. Bumstead of M. Ricord's annotations, and a reprint of Mr. Babington's edition, with notes and additions by the American editor. The additions to the original text are thus four-fold; and the usual editorial brackets present the rather startling array of interpolated names—"RICORD," "HOME," "EDITOR," and "G. C. B."

Exception may, we think, be not unreasonably taken, in some particulars, to this mosaic style of publication. But we must admit that an acceptable service has been performed in presenting this book to the profession. The observations of John Hunter on the Venereal Disease, must, as Mr. Babington justly observes, "always form an essential part of the study of every surgeon who wishes to make himself acquainted with the disease of which it treats;" and upon the value of the facts and opinions of Ricord, which are engrafted on it, we need not dwell. The notes and additions of the American editor are judicious and interesting. An index has been added, too, which cannot fail to increase the usefulness of the work.

